# Supplementary material for: Accuracy of the diagnosis of pneumonia in Canadian pediatric emergency departments: A prospective cohort study
Source: PLoS One. 2024 Dec 11;19(12):e0311201. doi: 10.1371/journal.pone.0311201 (PMC11633949; doi:10.1371/journal.pone.0311201)
Supplement: S2 File — (PDF) [file pone.0311201.s002.pdf]

Research Ethics Board (REB)

The Research Ethics Board for The Hospital for Sick Children is organized and operates according to the principles and practices outlined in the Tri-Council Policy Statement, the ICH Harmonized Tripartite Guidelines: Good Clinical Practice, and Division 5 and the Medical Devices Regulations of the Food and Drug Act as well as the Natural Health Products Regulations of Health Canada. This signed document is in lieu of the Health Canada Research Ethics Board Attestation Form.

Approval & Terms of Agreement

Investigators: Dr. Kathy Boutis, D.Johnson, J.Kellner, T.Klassen, A.Nettel-Aguirre, J.Williamson, T.Lynch, J.Fox, T.Marrie, M.Osmond, B.Muller, C.Slupsky, R.Brant, K.Fonseca

Study Title: Exploring novel methods to improve our diagnostic accuracy of childhood bacterial pneumonia

REB File number: 1000012424 **Level of Continuing Review:** II D
Protocol Version Date: Research Protocol – June 2008
Consent & Assent Form Version Date(s): Research Consent Form (Parent/Guardian) – January 23, 2008, Research Consent Form (Child) – July 2, 2008, Assent Form – June 19, 2008.
Investigator's Brochure Version Date: N/A
Other Approved Recruitment Document Dates: N/A

I agree to carry out the proposed research involving human subjects in accordance with the above-noted guidelines and regulations (as applicable) and using only the REB-approved study protocol and consent/assent form(s). I shall notify the division/department head and the REB prior to implementing any amendments in the protocol and consent/assent forms and of any deviations or any changes in study activity. I shall also notify the REB of any unexpected adverse events as per REB guidelines. As applicable, I certify that the research contract and corresponding protocol are consistent and will inform the contract manager of any protocol amendments as required.

I agree that, in accordance with the Personal Health Information Protection Act of Ontario, I am responsible for adhering to all conditions and restrictions imposed by the REB governing the use, security, disclosure, return and disposal of the research subjects' personal health information. I am also responsible for reporting immediately any privacy breaches to the REB Chair and to Janice Campbell, the Sick Kids privacy officer. I will ensure that the personal health information is used, only as necessary, to fulfill the specific research objectives and related research questions described in this application and approved by the REB.

Signature of Principal Investigator ______________________________DATE___________________

I approve of this research protocol, agree to share responsibility for its proper conduct, and will ensure that the REB is notified of concerns, as appropriate.

Signature of Division/Department Head _________________________DATE_____________________

The REB of the Hospital for Sick Children has reviewed and approved the above-named research study.

____

Mr. Richard Sugarman, REB Chair
555 University Avenue, Toronto, Ontario, M5G 1X8
Tel: 416-813-6152 Fax: 416-813-5085 Email: richard.sugarman@sickkids.ca

DATE OF APPROVAL ______________________________ EXPIRY DATE __________________
