# Supplementary material for: Accuracy of the diagnosis of pneumonia in Canadian pediatric emergency departments: A prospective cohort study
Source: PLoS One. 2024 Dec 11;19(12):e0311201. doi: 10.1371/journal.pone.0311201 (PMC11633949; doi:10.1371/journal.pone.0311201)
Supplement: S4 File — (PDF) [file pone.0311201.s004.pdf]

Office of Research Ethics

The University of Western Ontario
Room 00045 Dental Sciences Building, London, ON, Canada N6A 5C1
Telephone: (519) 661-3036 Fax: (519) 850-2466 Email: ethics@uwo.ca
Website: www.uwo.ca/research/ethics

Western

Use of Human Subjects - Ethics Approval Notice

Principal Investigator: Dr. T. Lynch
Review Number: 13700E **Review Level:** Expedited
Review Date: October 24, 2007
Protocol Title: Exploring Novel Methods to Improve our Diagnostic Accuracy of Childhood Bacterial Pneumonia
Department and Institution: Paediatrics, London Health Sciences Centre
Sponsor: CIHR-CANADIAN INSTITUTE OF HEALTH RESEARCH
Ethics Approval Date: November 20, 2007 **Expiry Date:** December 31, 2009
Documents Reviewed and Approved: UWO Protocol, Letter of Information and Consent.
Documents Received for Information:

This is to notify you that The University of Western Ontario Research Ethics Board for Health Sciences Research Involving Human Subjects (HSREB) which is organized and operates according to the Tri-Council Policy Statement: Ethical Conduct of Research Involving Humans and the Health Canada/ICH Good Clinical Practice Practices: Consolidated Guidelines; and the applicable laws and regulations of Ontario has reviewed and granted approval to the above referenced study on the approval date noted above. The membership of this REB also complies with the membership requirements for REB's as defined in Division 5 of the Food and Drug Regulations.

The ethics approval for this study shall remain valid until the expiry date noted above assuming timely and acceptable responses to the HSREB's periodic requests for surveillance and monitoring information. If you require an updated approval notice prior to that time you must request it using the UWO Updated Approval Request Form.

During the course of the research, no deviations from, or changes to, the protocol or consent form may be initiated without prior written approval from the HSREB except when necessary to eliminate immediate hazards to the subject or when the change(s) involve only logistical or administrative aspects of the study (e.g. change of monitor, telephone number). Expedited review of minor change(s) in ongoing studies will be considered. Subjects must receive a copy of the signed information/consent documentation.

Investigators must promptly also report to the HSREB:

a) changes increasing the risk to the participant(s) and/or affecting significantly the conduct of the study;
b) all adverse and unexpected experiences or events that are both serious and unexpected;
c) new information that may adversely affect the safety of the subjects or the conduct of the study.

If these changes/adverse events require a change to the information/consent documentation, and/or recruitment advertisement, the newly revised information/consent documentation, and/or advertisement, must be submitted to this office for approval.

Members of the HSREB who are named as investigators in research studies, or declare a conflict of interest, do not participate in discussion related to, nor vote on, such studies when they are presented to the HSREB.

[signature]

Chair of HSREB: Dr. John W. McDonald

Ethics Officer to Contact for Further Information			
☐ Janice Sutherland (jsutherland@uwo.ca)	☐ Jennifer McEwen (jmcewen4@uwo.ca)	☑ Grace Kelly (grace.kelly@uwo.ca)	☐ Denise Grafton (dgrafton@uwo.ca)

This is an official document. Please retain the original in your files.

cc: ORE File
LHRI
