# Supplementary material for: Accuracy of the diagnosis of pneumonia in Canadian pediatric emergency departments: A prospective cohort study
Source: PLoS One. 2024 Dec 11;19(12):e0311201. doi: 10.1371/journal.pone.0311201 (PMC11633949; doi:10.1371/journal.pone.0311201)
Supplement: S6 File — (PDF) [file pone.0311201.s006.pdf]

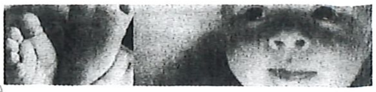

Sainte-Justine 1907-2007 à faire grandir la vie.

CHU Sainte-Justine
Le centre hospitalier universitaire mère-enfant

Pour l'amour des enfants

Université de Montréal

Le 13 mai 2008

Dr Serge Gouin
Urgence
Étage 1er Bloc 1

OBJET: Titre du projet: Exploring Novel Methods to Improve our Diagnostic Accuracy of Childhood Bacterial Pneumonia: A prospective cohort

No. de dossier: 2697

Responsables du projet: Serge Gouin M.D., chercheur responsable au CHU Sainte-Justine. Chercheur principal: Dr Tim Lynch, Children's Hospital of Western Ontario. Collaborateur: Dr David Johnson, Alberta Children's Hospital

Cher Docteur,

Votre projet cité en rubrique a été approuvé par le Comité d'éthique de la recherche en date d'aujourd'hui. Vous trouverez ci-joint la lettre d'approbation du Comité, la liste des documents approuvés ainsi que vos formulaires d'information et de consentement estampillés dont nous vous prions de vous servir d'une copie pour distribution.

Tous les projets de recherche impliquant des sujets humains doivent être réexaminés annuellement et la durée de l'approbation de votre projet sera effective jusqu'au 13 mai 2009. Notez qu'il est de votre responsabilité de soumettre une demande au Comité pour que votre projet soit renouvelé avant la date d'expiration mentionnée. Il est également de votre responsabilité d'aviser le Comité dans les plus brefs délais de toute modification au projet ainsi que de tout effet secondaire survenu dans le cadre de la présente étude.

Nous vous souhaitons bonne chance dans la réalisation de votre projet et vous prions de recevoir nos meilleures salutations.

Jean-Marie Therrien, Ph.D., éthicien
Président du Comité d'éthique de la recherche

JMT/sg

3175, Côte-Sainte-Catherine
Montréal (Québec)
H3T 1C5
www.chu-sainte-justine.org

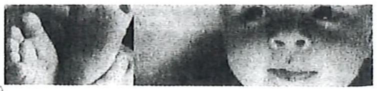

Sainte-Justine
1907-2007
à faire grandir la vie.

LE COMITÉ D'ÉTHIQUE DE LA RECHERCHE

Un comité du CHU Sainte-Justine formé des membres suivants:

CHU Sainte-Justine
Le centre hospitalier universitaire mère-enfant

Pour l'amour des enfants

Université de Montréal

Jean-Marie Therrien, éthicien et président

Anne-Claude Bernard-Bonnin, pédiatre

Geneviève Cardinal, juriste

Marie Saint-Jacques, infirmière de recherche

Françoise Grambin, représentante du public

Albert Moghrabi, hémato-oncologue

Lyne Pedneault, pharmacienne

Andrea Richter, scientifique

Ragnhild Milewski-Laporte, représentante du public

Les membres du comité d'éthique de la recherche ont étudié le projet de recherche clinique intitulé:

Exploring Novel Methods to Improve our Diagnostic Accuracy of Childhood Bacterial Pneumonia: A prospective cohort

No. de dossier: 2697

soumis par: *Serge Gouin M.D., chercheur responsable au CHU Sainte-Justine. Chercheur principal: Dr Tim Lynch, Children's Hospital of Western Ontario. Collaborateur: Dr David Johnson, Alberta Children's Hospital*

et l'ont trouvé conforme aux normes établies par le comité d'éthique de la recherche du CHU Sainte-Justine. Le projet est donc accepté par le Comité.

Jean-Marie Therrien, Ph.D., éthicien
Président du Comité d'éthique de la recherche

Date d'approbation: 13 mai 2008

3175, Côte-Sainte-Catherine
Montréal (Québec)
H3T 1C5
www.chu-sainte-justine.org

Sainte-Justine 1907-2007 à faire grandir la vie.

CHU Sainte-Justine
Le centre hospitalier universitaire mère-enfant

Pour l'amour des enfants

Université de Montréal

Liste des documents approuvés par le CER

Titre du projet:
Exploring Novel Methods to Improve our Diagnostic Accuracy of Childhood Bacterial Pneumonia: A prospective cohort

No. de dossier: 2697

Date d'approbation : mardi le 13 mai 2008

Responsables du projet: Serge Gouin M.D., chercheur responsable au CHU Sainte-Justine. Chercheur principal: Dr Tim Lynch, Children's Hospital of Western Ontario. Collaborateur: Dr David Johnson, Alberta Children's Hospital

- Brochure de l'investigateur datée ☐
- Protocole de recherche daté du ~ Septembre 2007 ☒
- Formulaire(s) de consentement daté(s)
  - Français (3 avril 2008) ☒
  - Anglais (20 février 2008) ☒
- Publicité en vue du recrutement ☐
- Lettre d'information aux participants ☐
- Questionnaires ☐
- Politique de gestion de banque de données datée du ☐
- Autre ☐
  Préciser : ____________________

3175, Côte-Sainte-Catherine
Montréal (Québec)
H3T 1C5
www.chu-sainte-justine.org
