# Supplementary material for: Accuracy of the diagnosis of pneumonia in Canadian pediatric emergency departments: A prospective cohort study
Source: PLoS One. 2024 Dec 11;19(12):e0311201. doi: 10.1371/journal.pone.0311201 (PMC11633949; doi:10.1371/journal.pone.0311201)
Supplement: S7 File — (PDF) [file pone.0311201.s007.pdf]

Health Research Ethics Board

308 Campus Tower
University of Alberta, Edmonton, AB T6G 1K8
p. 780.492.9724 (Biomedical Panel)
p. 780.492.0302 (Health Panel)
p. 780.492.0459

Re-Approval Form

Date:	October 26, 2010
Principal Investigator:	Sarah Curtis
Renewal ID:	Pro00002063_REN3
Study ID:	Pro00002063
Study Title:	Exploring novel methods to improve our diagnostic accuracy of childhood bacterial pneumonia
Approval Expiry Date:	December 7, 2011
Sponsor/Funding Agency:	CIHR - Canadian Institutes for Health Research

The Health Research Ethics Board - Biomedical Panel has reviewed the renewal request and file for this project and found it to be acceptable within the limitations of human experimentation.

The re-approval for the study as presented is valid for one year. It may be extended following completion of the annual renewal request. Beginning 45 days prior to expiration, you will receive notices that the study is about to expire. Once the study has expired you will have to resubmit. Any proposed changes to the study must be submitted to the HREB for approval prior to implementation.

All study-related documents should be retained, so as to be available to the HREB on request. They should be kept for the duration of the project and for at least five years following study completion.

Sincerely,

S.K.M. Kimber, MD, FRCPC
Chair, Health Research Ethics Board - Biomedical Panel

Note: This correspondence includes an electronic signature (validation and approval via an online system).

UNIVERSITY OF ALBERTA   Alberta Health Services   Covenant Health
