# Supplementary material for: Accuracy of the diagnosis of pneumonia in Canadian pediatric emergency departments: A prospective cohort study
Source: PLoS One. 2024 Dec 11;19(12):e0311201. doi: 10.1371/journal.pone.0311201 (PMC11633949; doi:10.1371/journal.pone.0311201)
Supplement: S1 File — (PDF) [file pone.0311201.s001.pdf]

FACULTY OF MEDICINE | UNIVERSITY OF CALGARY

**OFFICE OF MEDICAL BIOETHICS**
Room 93, Heritage Medical Research Bldg
3330 Hospital Drive NW
Calgary, AB, Canada T2N 4N1
Telephone: (403) 220-7990
Fax: (403) 283-8524
Email: omb@ucalgary.ca

2008-02-14

Dr. David W. Johnson
Department of Paediatrics
Alberta Children's Hospital
Calgary, Alberta

**Dear Dr. Johnson:**

**RE: Exploring Novel Methods to Improve our Diagnostic Accuracy of Childhood Bacterial Pneumonia**

**Ethics ID: E-21505**

The above-named research, including the Consent Form (version 1.0, November 27, 2007 ), Ethics Approval Form (University of Western Ontario, November 20, 2007 ), Protocol (September 2007 ), CIHR Reviews has been granted ethical approval by the Conjoint Health Research Ethics Board of the Faculties of Medicine, Nursing and Kinesiology, University of Calgary, and the Affiliated Teaching Institutions. The Board conforms to the Tri-Council Guidelines, ICH Guidelines and amendments to regulations of the Food and Drugs Act re clinical trials, including membership and requirements for a quorum.

You and your co-investigators are not members of the CHREB and did not participate in review or voting on this study.
Please note that this approval is subject to the following conditions:
(1) appropriate procedures for consent for access to identified health information have been approved;
(2) a copy of the informed consent form must have been given to each research subject, if required for this study;
(3) a Progress Report must be submitted by **February 14, 2009**, containing the following information:
- i) the number of subjects recruited;
- ii) a description of any protocol modification;
- iii) any unusual and/or severe complications, adverse events or unanticipated problems involving risks to subjects or others, withdrawal of subjects from the research, or complaints about the research;
- iv) a summary of any recent literature, finding, or other relevant information, especially information about risks associated with the research;
- v) a copy of the current informed consent form;
- vi) the expected date of termination of this project.

4) a Final Report must be submitted at the termination of the project.

Please accept the Board's best wishes for success in your research.
Yours sincerely,

L. McGovern

~~Liz~~ M[c]Govern
Ethics Resource Officer
For
Glenys Godlovitch, BA(Hons), LLB, PhD
Chair, Conjoint Health Research Ethics Board

GG/emcg
c.c. Child Health Research Office   Ms. Gladys Glowacki (Health Records)   Ms. Donna McDonald (RTA)   Dr. F. belanger(information)   Research Services  Ms. C.C. Victorino (Research Coordinator)
Office of Information & Privacy
