# Supplementary material for: Accuracy of the diagnosis of pneumonia in Canadian pediatric emergency departments: A prospective cohort study
Source: PLoS One. 2024 Dec 11;19(12):e0311201. doi: 10.1371/journal.pone.0311201 (PMC11633949; doi:10.1371/journal.pone.0311201)
Supplement: S3 File — (PDF) [file pone.0311201.s003.pdf]

**QUEEN'S UNIVERSITY HEALTH SCIENCES & AFFILIATED TEACHING HOSPITALS RESEARCH ETHICS BOARD**

Queen's UNIVERSITY

March 14, 2008

This Ethics Application was subject to:

☒ Full Board Review
Meeting Date: February 11, 2008
☐ Expedited Review

Dr. R. van Wylick
Department of Paediatrics
Brock 3, Room 382
Hotel Dieu Hospital

Dear Dr. vanWylick,

| | |
|---|---|
| **Study Title:** | **Exploring Novel Methods to Improve Our Diagnostic Accuracy of Childhood Bacterial Pneumonia** |
| **Co-Investigators:** | **Dr. T. Lynch** |

The members of the Queen's University Health Sciences & Affiliated Teaching Hospitals Research Ethics Board have examined the protocol (September 2007), Intake Form (August 2007) and revised consent form (V2 - February 25, 2008) and revised Assent Form (February 29, 2008) for your project (as stated above) and consider it to be ethically acceptable. This approval is valid for one year from the date of the Chair's signature below. Please attend carefully to the following list of ethics requirements you must fulfill over the course of your study:

- **Reporting of Amendments**: If there are any changes to your study (e.g. consent, protocol, study procedures, etc.), you must submit an amendment to the Research Ethics Board for approval. (see http://www.queensu.ca/vpr/reb.htm).

- **Reporting of Serious Adverse Events**: Any unexpected serious adverse event occurring locally must be reported within 2 working days or earlier if required by the study sponsor. All other serious adverse events must be reported within 15 days after becoming aware of the information.

- **Reporting of Complaints**: Any complaints made by participants or persons acting on behalf of participants must be reported to the Research Ethics Board within 7 days of becoming aware of the complaint. <u>Note</u>: All documents supplied to participants must have the contact information for the Research Ethics Board.

- **Annual Renewal**: Prior to the expiration of your approval (which is one year from the date of the Chair's signature below), you will be reminded to submit your renewal form along with any <u>new</u> changes or amendments you wish to make to your study. If there have been no major changes to your protocol, your approval may be renewed for another year.

Yours sincerely,

Albert Clark
Chair, Research Ethics Board

March 17, 2008
Date

ORIGINAL TO INVESTIGATOR - COPY TO DEPARTMENT HEAD- COPY TO HOSPITAL(S) /P&T (If appropriate) - FILE COPY

**Study Code: PAED-211-08**

- **Investigators please note that if your trial is registered by the sponsor, you must take responsibility to ensure that the registration information is accurate and complete**

**QUEEN'S UNIVERSITY HEALTH SCIENCES & AFFILIATED TEACHING HOSPITALS RESEARCH ETHICS BOARD**

Queen's UNIVERSITY

**The membership of this Research Ethics Board complies with the membership requirements for Research Ethics Boards as defined by the Tri-Council Policy Statement; Part C Division 5 of the Food and Drug Regulations, OHRP, and U.S DHHS Code of Federal Regulations Title 45, Part 46 and carries out its functions in a manner consistent with Good Clinical Practices.**

**Federalwide Assurance Number : #FWA00004184**
**#IRB00001173**

**Current membership of the Queen's University Health Sciences & Affiliated Teaching Hospitals Research Ethics Board**

| | |
|---|---|
| **Dr. A.F. Clark** | **Emeritus Professor, Department of Biochemistry, Faculty of Health Sciences, Queen's University (Chair)** |
| **Dr. H. Abdollah** | **Professor, Department of Medicine, Queen's University** |
| **Rev. T. Deline** | **Community Member** |
| **Dr. M. Evans** | **Community Member** |
| **Dr. S. Irving** | **Psychologist, Providence Care, St. Mary's of the Lake Hospital Site** |
| **Prof. L. Keeping-Burke** | **Assistant Professor, School of Nursing, Queen's University** |
| **Dr. J. Low** | **Emeritus Professor, Department of Obstetrics and Gynaecology, Queen's University and Kingston General Hospital** |
| **Dr. W. Racz** | **Emeritus Professor, Department of Pharmacology & Toxicology, Queen's** |
| **Dr. H. Richardson** | **Assistant Professor, Department of Community Health & Epidemiology<br>Project Coordinator, NCIC CTG, Queen's University** |
| **Dr. B. Simchison** | **Assistant Professor, Department of Anesthesiology, Queen's University** |
| **Dr. A.N. Singh** | **WHO Professor in Psychosomatic Medicine and Psychopharmacology<br>Professor of Psychiatry and Pharmacology<br>Chair and Head, Division of Psychopharmacology, Queen's University<br>Director & Chief of Psychiatry, Academic Unit, Quinte Health Care, Belleville General Hospital** |
| **Dr. E. Tsai** | **Assistant Professor, Department of Paediatrics and Office of Bioethics, Queen's University** |
| **Rev. J. Warren** | **Community Member** |
| **Ms. K. Weisbaum** | **LL.B. and Adjunct Instructor, Department of Family Medicine (Bioethics)** |
| **Dr. S. Wood** | **Director, Office of Research Services (Ex-Officio)** |

**FACULTY OF HEALTH SCIENCES AND AFFILIATED TEACHING HOSPITALS DATA SHEET FOR RESEARCH INVOLVING HUMAN SUBJECTS**

**To be completed only if the research involves the use of human subjects**

**Attach to** Office Of Research Services, Research Data Summary And Signature Sheet

Ref: Ethics to Human Experimentation, Medical Research Guidelines 1997
Queen's University Health Sciences & Affiliated Teaching Hospitals Human Research Ethics Board Guidelines, 1993

**Project Title**: Exploring Novel Methods to Improve our Diagnostic Accuracy of Childhood Bacterial Pneumonia

1. Human subjects will be required to participate: NO [ ] YES [ X ]

2. The protocol has been submitted for ethics review: NO [ ] YES [ X ]

3. If answer to 1 is "YES" and 2 is "NO", please explain: ______________________________

4. Does this research require the participation of hospital patients? NO [ ] YES [ X ]

   Circle hospital(s) concerned: KGH (HDH) SMOL KPH Other (specify) ____________

5. Will hospital facilities be required for research involving human subjects? NO [ ] YES [X ]

   Circle hospital(s) concerned: KGH (HDH) SMOL KPH Other (specify) ____________

6. Will patients be selected from department other than your department of primary affiliation? If yes, your signature below indicates that you have received written consent from the Head[s] of hospital department[s] NO [ X ] YES [ ]

7. Do all research personnel having contact with patients have appropriate hospital Department appointments (i.e. staff, residents, departmental assistants)? NO [ ] YES [ X ]

8. Will research involve the use of hospital facilities over and above those required for normal patient care? NO [ X ] YES [ ]

| If "YES", indicate department: | Cost | Costs confirmed with (name of person) |
|---|---|---|
| Laboratory | | |
| Nursing | | |
| Pharmacy | | |
| Radiology | | |
| Other [please specify] | | |
| TOTAL | | |

Are these costs included in the budget of the grant application? NO [ ] YES [ ]
If not, how will they be paid for? ______________________________

[signature] Jan 11/08
Investigator Date

[signature] Jan 11/08
Department Head Date

Albert Clark
Chair, Research Ethics Board

**QUEEN'S UNIVERSITY HEALTH SCIENCES & AFFILIATED TEACHING HOSPITALS RESEARCH ETHICS BOARD**

March 14, 2008

This Ethics Application was subject to:

☒ Full Board Review
Meeting Date: February 11, 2008

☐ Expedited Review

Dr. R. van Wylick
Department of Paediatrics
Brock 3, Room 382
Hotel Dieu Hospital

Dear Dr. vanWylick,

**Study Title:** **Exploring Novel Methods to Improve Our Diagnostic Accuracy of Childhood Bacterial Pneumonia**

**Co-Investigators:** **Dr. T. Lynch**

The members of the Queen's University Health Sciences & Affiliated Teaching Hospitals Research Ethics Board have examined the protocol (September 2007), Intake Form (August 2007) and revised consent form (V2 - February 25, 2008) and revised Assent Form (February 29, 2008) for your project (as stated above) and consider it to be ethically acceptable. This approval is valid for one year from the date of the Chair's signature below. Please attend carefully to the following list of ethics requirements you must fulfill over the course of your study:

- **Reporting of Amendments**: If there are any changes to your study (e.g. consent, protocol, study procedures, etc.), you must submit an amendment to the Research Ethics Board for approval. (see http://www.queensu.ca/vpr/reb.htm).

- **Reporting of Serious Adverse Events**: Any unexpected serious adverse event occurring locally must be reported within 2 working days or earlier if required by the study sponsor. All other serious adverse events must be reported within 15 days after becoming aware of the information.

- **Reporting of Complaints**: Any complaints made by participants or persons acting on behalf of participants must be reported to the Research Ethics Board within 7 days of becoming aware of the complaint. Note: All documents supplied to participants must have the contact information for the Research Ethics Board.

- **Annual Renewal**: Prior to the expiration of your approval (which is one year from the date of the Chair's signature below), you will be reminded to submit your renewal form along with any new changes or amendments you wish to make to your study. If there have been no major changes to your protocol, your approval may be renewed for another year.

Yours sincerely,

Albert Clark
Chair, Research Ethics Board

March 17, 2008
Date

ORIGINAL TO INVESTIGATOR - COPY TO DEPARTMENT HEAD- COPY TO HOSPITAL(S) /P&T (If appropriate) - FILE COPY

**Study Code: PAED-211-08**

- **Investigators please note that if your trial is registered by the sponsor, you must take responsibility to ensure that the registration information is accurate and complete**

## QUEEN'S UNIVERSITY HEALTH SCIENCES & AFFILIATED TEACHING HOSPITALS RESEARCH ETHICS BOARD

Queen's UNIVERSITY

**The membership of this Research Ethics Board complies with the membership requirements for Research Ethics Boards as defined by the Tri-Council Policy Statement; Part C Division 5 of the Food and Drug Regulations, OHRP, and U.S DHHS Code of Federal Regulations Title 45, Part 46 and carries out its functions in a manner consistent with Good Clinical Practices.**

**Federalwide Assurance Number : #FWA00004184**
**#IRB00001173**

**Current membership of the Queen's University Health Sciences & Affiliated Teaching Hospitals Research Ethics Board**

| | |
|---|---|
| Dr. A.F. Clark | Emeritus Professor, Department of Biochemistry, Faculty of Health Sciences, Queen's University (Chair) |
| Dr. H. Abdollah | Professor, Department of Medicine, Queen's University |
| Rev. T. Deline | Community Member |
| Dr. M. Evans | Community Member |
| Dr. S. Irving | Psychologist, Providence Care, St. Mary's of the Lake Hospital Site |
| Prof. L. Keeping-Burke | Assistant Professor, School of Nursing, Queen's University |
| Dr. J. Low | Emeritus Professor, Department of Obstetrics and Gynaecology, Queen's University and Kingston General Hospital |
| Dr. W. Racz | Emeritus Professor, Department of Pharmacology & Toxicology, Queen's |
| Dr. H. Richardson | Assistant Professor, Department of Community Health & Epidemiology<br>Project Coordinator, NCIC CTG, Queen's University |
| Dr. B. Simchison | Assistant Professor, Department of Anesthesiology, Queen's University |
| Dr. A.N. Singh | WHO Professor in Psychosomatic Medicine and Psychopharmacology<br>Professor of Psychiatry and Pharmacology<br>Chair and Head, Division of Psychopharmacology, Queen's University<br>Director & Chief of Psychiatry, Academic Unit, Quinte Health Care, Belleville General Hospital |
| Dr. E. Tsai | Assistant Professor, Department of Paediatrics and Office of Bioethics, Queen's University |
| Rev. J. Warren | Community Member |
| Ms. K. Weisbaum | LL.B. and Adjunct Instructor, Department of Family Medicine (Bioethics) |
| Dr. S. Wood | Director, Office of Research Services (Ex-Officio) |
