# Supplementary material for: Accuracy of the diagnosis of pneumonia in Canadian pediatric emergency departments: A prospective cohort study
Source: PLoS One. 2024 Dec 11;19(12):e0311201. doi: 10.1371/journal.pone.0311201 (PMC11633949; doi:10.1371/journal.pone.0311201)
Supplement: S5 File — (PDF) [file pone.0311201.s005.pdf]

L'Hôpital de Montréal pour enfants
The Montreal Children's Hospital

Centre universitaire de santé McGill
McGill University Health Centre

Nov. 6, 2008

Dr. M. Bhatt
Emergency Medicine
Montreal Children's Hospital
Room T-124

Re: **PED-08-032 Exploring Novel Methods to Improve our Diagnostic Accuracy of Childhood Bacterial Pneumonia**
Funded by: **CIHR**

Dear Dr. Bhatt,

The above-named research proposal received Full Board review at the convened meeting of the Montreal Children's Hospital Research Ethics Board on August 25, 2008 and was found to be within ethical guidelines for conduct at the McGill University Health Centre, and was entered accordingly into the minutes of the Research Ethics Board (REB) meeting. At the MUHC, sponsored research activities that require US federal assurance are conducted under Federal Wide Assurance (FWA) 00000840.

We are pleased to inform you that final approval was provided on Sept. 30, 2008 for the following:

- research protocol, June 2008
- consent document (English & French version Sept. 29, 2008)
- assent document (English & French version Sept. 29, 2008)

All research involving human subjects requires review at a recurring interval and the current study approval is in effect until August 24, 2009 (anniversary of initial review). It is the responsibility of the principal investigator to submit an Application for Continuing Review to the REB prior to the expiration of approval to comply with the regulation for continuing review of "at least once per year".

It is important to note that an MUHC translator has certified the validation of the translated version of the consent and assent document. As the translated text was potentially modified, the document must be reviewed by the study sponsor prior to its use. Any further modification to the REB approved and certified consent document must be identified by a revised date in the document footer, and re-submitted for review prior to its use.

The Research Ethics Boards (REBs) of the McGill University Health Centre are registered REBs working under the published guidelines of the Tri-Council Policy Statement, in compliance with the "Plan d'action ministériel en éthique de la recherche et en intégrité scientifique" (MSSS, 1998) and the Food and Drugs Act (7 June, 2001), acting in conformity with standards set forth in the (US) Code of Federal Regulations governing human subjects research, and functioning in a manner consistent with internationally accepted principles of good clinical practice.

We wish to advise you that this document completely satisfies the requirement for Research Ethics Board Attestation as stipulated by Health Canada.

The project was assigned MUHC Study Number **PED-08-032** that is required as MUHC reference when communicating about the research. Should any revision to the study, or other unanticipated development occur prior to the next required review, you must advise the REB without delay. Regulation does not permit initiation of a proposed study modification prior to REB approval for the amendment.

Sincerely,

Jane McDonald, M.D., F.R.C.P.C
Chairperson, Research Ethics Board
