# Supplementary material for: Accuracy of the diagnosis of pneumonia in Canadian pediatric emergency departments: A prospective cohort study
Source: PLoS One. 2024 Dec 11;19(12):e0311201. doi: 10.1371/journal.pone.0311201 (PMC11633949; doi:10.1371/journal.pone.0311201)
Supplement: Attachment — Submitted filename: RESPONSE TO EDITOR AND REVIEWERS.docx [file pone.0311201.s008.docx]

**RESPONSE TO THE ORIGINAL REVIEWERS' COMMENTS**

Our previously submitted manuscript, “Accuracy of the diagnosis of pneumonia in the pediatric Emergency Department: a prospective cohort study” [PONE-D-23-41978], was never sent to reviewers for assessment. Rather our manuscript was originally rejected because, at that time, we did not have copies of the original Research Ethics approval letter for two of the seven Canadian pediatric emergency departments who participated in our study. Through re-doubling our efforts, we have now located the original Research Ethics Board approval letters for the two sites which were previously missing. Copies of all seven Research Ethics approval letters have been uploaded with this resubmission. In the PLOS ONE decision response (PLOS ONE decision email copied below with key sentences/phrases highlighted in yellow), the only concern raised was our inability to provide Research Ethics approval letters for all seven sites.

Because our manuscript was never sent to reviewers, we did not receive any comments or suggestions for change to our manuscript. Hence we did not need to make any changes to the manuscript. Nonetheless we have attached a *Tracked Changes Word Document* comparing the last submitted manuscript for the prior *PONE-D 23-41978* submission with the manuscript submitted with this document. You will note we have made a few minor formatting and term changes; there are no substantive changes.

PONE-D-23-41978
Accuracy of the diagnosis of pneumonia in the pediatric Emergency Department: a prospective cohort study
PLOSONE

Dear Dr. Robinson,

Thank you very much for submitting your manuscript to PLOS ONE, and for responding to our recent requests regarding your submission. After careful evaluation, we have decided that your submission does not meet our publication criteria and must be rejected.

Based on our evaluation of your manuscript and the information you provided, we do not feel that your submission meets our ethical requirements for human subjects research submissions. PLOS ONE requires that research meets all applicable standards for the ethics of experimentation and research integrity (<http://journals.plos.org/plosone/s/human-subjects-research>). We reserve the right to reject any submission that does not meet our internal ethical standards, which in some cases are more stringent than local ethical standards.

You have not submitted the requested ethics approval documents and we cannot accept retrospective personal assurance letters in place of the required documents. It is therefore not clear whether you obtained the necessary ethical approval for the study to take place. Please be aware that we expect all research involving human participants and/or medical data to have been approved by the authors' Institutional Review Board (IRB) or by equivalent ethics committee(s).

Since you did not obtain the necessary approvals to conduct this research we are unable to consider your manuscript. I am sorry that we cannot be more positive on this occasion, but I hope you appreciate the reasons for this decision.

Yours sincerely,

KalaraniAnbarasan
EditorialOffice
PLOS ONE
