# Supplementary material for: Accuracy of the diagnosis of pneumonia in Canadian pediatric emergency departments: A prospective cohort study
Source: PLoS One. 2024 Dec 11;19(12):e0311201. doi: 10.1371/journal.pone.0311201 (PMC11633949; doi:10.1371/journal.pone.0311201)
Supplement: Attachment — Submitted filename: RESPONSE TO REVIEWERS.docx [file pone.0311201.s009.docx]

**RESPONSE TO REVIEWERS**

We appreciate the opportunity to revise our manuscript to meet the reviewers concerns and comments.

Reviewer #1: Thanks for asking me to review this interesting paper. I have some minor comments as below:

1. Title:
My understanding is the study focused on Paed ED in high income countries. Better to include that information in title since the approach in "low income", "middle income" is somewhat different.

**Response: Thanks for this good suggestion. We changed the title to “*Accuracy of the diagnosis of pneumonia in Canadian pediatric emergency departments: a prospective cohort study*”.**

2. Introduction

3. Method
I am not really clear how the Expert Consensus made decisions of "no pneumonia", "bacterial pneumonia", "atypical pneumonia", "viral pneumonia". Did they use any specific cut off for CBC, CRP, PCT, ESR? Also, what changes on CXR would suggest "atypical pneumonia", "typical pneumonia", "viral pneumonia".

**Response: The panelists used their own expertise to make all decisions. We then used their decisions to determine cut-offs for laboratory values. To clarify this, we changed/added the following sentences:**

**Line 84 – *A panel of experienced pediatric sub-specialists reviewed each case using their individual clinical expertise to achieve consensus.***

**Line 134 - *Each panelist used their clinical expertise to interpret the significance of laboratory test results and chest radiographs; no interpretation standards were imposed on panelists.***

A common practice in ED is children > 5 years old would be covered both "bacterial pneumonia" and "atypical bacterial pneumonia". Did you record these cases in your study?

**Response: We agree that this is a common practice but the focus of this study was on diagnosis rather than treatment so we do not have the requested data.**

4. Results
- Table 1: please document unit that belongs to each factor. Ex: O2 sat room air (%), HR (bpm), fever (>=38) (n)

**Response: Units were added to all tables. Thanks for noticing this deficiency.**

- Table 2: what is the subgroup of "no pneumonia" + blood culture positive?

**Response: The three cases judged by the panel not to be pneumonia with positive blood cultures grew *Streptococcus pneumoniae*. Lines 248 and 249 – the following was added to Table 1, Footnote 3. “*Blood cultures were positive for Streptococcus pneumoniae (n=5 (2 “Bacterial Pneumonia/ 3 “No Pneumonia”), Staphylococcus aureus (N=1 (“Bacterial Pneumonia”) and Gram-negative bacilli (N=1 (“Bacterial Pneumonia”))*”**

How many cases NPS positive + superimposed infection?

**Response – Table 2 shows that 17 children with bacterial pneumonia also had a virus detected.**

- Table 4:
Why to choose the cut off 4, 15 for WBC? 42 for CRP? 47 for ESR? 0.85 for PCT? Any references?

**Response: As stated in the methods section, “*For ESR, CRP and PCT, optimal cut-points were determined using area under the Receiver Operation Curve (ROC) and logistic regression modeling. These cut-points were then used to predict each laboratory test’s diagnostic accuracy for bacterial pneumonia*.” We used WBC cutoffs standardly used in clinical practice and the published literature.**

Please add a foot note for abbreviations: MD, EN (*we presume the reviewer meant RN*)

**Response: Footnote was added for MD (medical doctor) and RN (registered nurse).**

5. Discussion
My main concern is how to apply the "Expert consensus" in practice as children present to ED require an urgent approach and management. Dx of pneumonia is based on clinical findings.

**Response: We completely agree. The expert panel was convened to derive practical tips for diagnosis of pneumonia that can be used by clinicians in the future. We did not intend that other centers would set up a similar expert panel.**

Reviewer #2: Thank you for opportunity to review. Well executed project with large sample and good design. The manuscript is well written. No changes to suggest. Would recommend it is ready for publication in its current form

Reviewer #3: Line 52-54: requires a reference.

**Response: We changed the sentence to read: “*For typical bacterial pneumonia, antibiotics appear to hasten resolution, prevent almost all deaths, and may decrease the incidence of complications including empyema or abscess.*” As far as we know, the efficacy of antibiotics in pediatric bacterial pneumonia has never been proven.**

Line 117-123: Mentions the parental logs; it may be useful to include how completely these were filled. What training did the parents receive?

**Response: The only parent log for the study was the fever diary. Parents were given a fever diary to fill out for 7 days after discharge from ED. This was reviewed with them by the nurse that enrolled them, and was included on the enrolment checklist of things to complete with the family before they were discharged. There were 11 cases that we were unable to contact after they were discharged (lost to follow-up), and another 31 that did not complete the fever diary. We added the following sentence starting on line 228 to the Results section: *Of these 247 cases, 11 could not be contacted for follow-up and another 31 did not complete the fever diary but provided the other follow-up data.***

Methods: why not also stratify the consensus diagnosis into non pneumonia cases? It may have been useful to know how many of cases which were incorrectly diagnosed as pneumonia were bronchiolitis, asthma etc.

**Response: We did not collect data on the final diagnosis of non-pneumonia cases as this fell outside of the objectives of our study. It would have required much more extensive follow-up to reliably establish the final diagnosis in non-pneumonia cases.**

By limiting the consensus diagnosis certainty to >50% it removes the ability of experts who still disagreed to reflect that, or to show that they were very uncertain and it means that the certainty on the censuses diagnosis of 79 is not really comparable to the others where the experts could choose between 0 and 100. It artificially inflates this certainty.

**Response: To better clarify how the panelists were able to express a lack of certainty, we added the following sentence starting on line 185 to the methods section: Certainty was determined by calculating the percentage difference between the chosen value and 50.0%, then this number was multiplied by 2. This allowed certainty to range from a minimum of 0 to a maximum of 100. For example, if a panelist chose 51.0%, then the calculated certainty would be 2.0%,**

Results:
Lines 218-222: Enrolling only 269 out of 1294 eligible patients means there is room that a lot of information was missed. Were there any trends to who which children were most likely to decline consent i.e. more unwell children, first nations children, those presenting at night etc. Could these be associated with worse clinically outcomes which limited the generally applicability of your results?

**Response: We agree that this is one of the limitations of our study. However, unfortunately we could not collect demographic data on those who were not enrolled. We added the following sentence starting on line 370 to the discussion: “*A fourth limitation is that because of the relatively small number of children enrolled and reported on (247) as compared with those who met study eligibility (1,294) and that we do not have demographic and clinical details of those not enrolled, it is possible that our cohort of children does not accurately represent the full range of all children suspected of having pneumonia.”***

Table 1 and 2: These tables are a little bit difficult to know which statistics are the best representation of data spread for each variable. Most of these characteristics and results were given to us as both medians + IQR and means +SD. If a variable is parametric just report on Mean and SD and if it is non parametric then a median and IQR. By giving us both for every characteristic it makes it more difficult for a reader to know what information is actually the most appropriate descriptor of spread for that variable.

**Response: We thank the reviewer for pointing this out and have simplified the tables as suggested.**

I also would have been very interested to see a table breaking down how the expert examples at different parts of the diagnosis process compared to the ED physicians.

**Response: While it is possible to construct these additional tables, we worry that providing these additional data would be confusing to readers. If the editor disagrees with us and believes it is important to add this table, we will do so.**

Table 2: I found it interesting that 30% of the bacterial pneumonias had entero/rhino present. Is this worth commenting on. I often see junior doctors falsely reassured by the presence of entero/rhino virus on an NPA.

**Response: We added the following sentence starting on line 347 to the discussion: “*Our data also show that detection of viruses does not exclude bacterial pneumonia as almost one-third of those with bacterial pneumonia had viral coinfection.”***

Table 3:
You mention that this table demonstrated discrepancies, but this table actually shows how often each group (ED or Consensus) make each diagnosis, but not how often they agree and disagree. Your Sankey diagram is what shows the actual discrepancies. For the table to do this this you would need to create a 5x5 table with one Axis listing the ED MD diagnosis and the other listing Consensus diagnosis so you can see how often they were in agreement.

**Response: We agree with the reviewer that our Sankey diagram shows the ‘actual discrepancies’ between ED physicians and the Consensus Panel, and that Table 3 highlights overall differences in frequency and percentage of diagnoses. That we why we titled the table: “*Differences in Frequency and Percentage for ED physician diagnoses versus Panel Consensus Diagnosis for children assessed in the ED with cough and fever who had a CXR performed*”.**

Results and conclusion:
I think it would be worth a more nuanced approach to conclusions than p<0.05 = absolutely significant and p>0.05 means no significance. Clinical relevance could be discussed more. i.e. lines 323-325 you state that fever was not associated with a diagnosis. Whilst true in the most rigid sense, you actually did find a trend associating with fever and the presence of typical bacterial pneumonia, however it missed out on statistical significance by a narrow margin (p=0.0524). This would have been a reasonable part of the paper to mention that statistical significance is not the same as clinical relevance. A p of 0.0524 compared to a p of 0.0499 is completely arbitrary and fever is still likely a useful factor in discriminating typical bacterial pneumonia from other causes. The same is true of wheeze, the way you talked about it with it being significant for one type of pneumonia if heard by a nurse, but the other if heard by a physician is not very clinically relevant. For both typical and overall pneumonia, absence of wheeze from nurses and physicians either reached significance or was trending close to significance. I also wondered if it was worth grouping those two findings as just wheeze heard on examination by a either study nurse or physician, as I think both professionals findings are relevant and wheeze can be an intermittent finding.

**Response: We agree with the reviewer and have modified the discussion to reflect their points about both the ‘significance’ of fever and wheeze. With regard to fever, starting on line 339, we wrote the following: “*It is important to note, however, that fever on admission just missed achieving statistical significance (p = 0.0524). The borderline significance of this finding may be due to our inclusion criteria requiring children to have either a history or presence of fever to be enrolled.”* With regard to wheeze observed by a nurse versus a physician, on line 330, we wrote: “*The significance of wheeze detected by the nurse versus the physician is unclear as wheeze can be intermittent.*”**

The way the over/under 5 statement is framed feels a bit incomplete. I understand that you are saying that the pretest probability of pneumonia in an over 5 year old presenting to ED is higher, but I think it would be worth at least flagging that the majority of pneumonias were in children under 5 (and possibly higher for typical pneumonia, but we were not given that information). The way this was written feels like a junior doctor reading it could think that pneumonia is less common in 5 year old, rather than what this actually says which is that 5 year olds present much more often with these symptoms in general, but a similar number of them overall (32 v 30 in your findings) will have have pneumonia.

**Response: Thanks for pointing out that our former wording was misleading. We now have added the following sentence starting on line 329: *However, it is of note that the majority of children with bacterial pneumonia were 5 years of age or younger.***

Lastly, I think you could give ED physicians a little bit more credit. I think it is probably worth giving them some credit for the fact that of the discrepancies there were almost no missed cases of TBP. Looking at the Sankey I think maybe one misdiagnosed as NP and one misdiagnosed as ABP. This means there were almost no children placed at significant risk of adverse outcome due to undertreated pneumonia, but we could be better with our antimicrobial stewardship. I actually read that as the ED physicians doing pretty good job. I am not worried about the difference between viral pneumonia and no pneumonia, as the treatment is so similar for the most part.

**Response: We completely agree with this point and added the following sentence to the first paragraph, starting on line 322, of the discussion: *ED physicians achieved this goal as they diagnosed bacterial pneumonia for all but one of 62 cases that the panelists considered to be bacterial pneumonia.***
