# Supplementary material for: Accuracy of the diagnosis of pneumonia in Canadian pediatric emergency departments: A prospective cohort study
Source: PLoS One. 2024 Dec 11;19(12):e0311201. doi: 10.1371/journal.pone.0311201 (PMC11633949; doi:10.1371/journal.pone.0311201)
Supplement: Attachment — Submitted filename: Response to Reviewers,Sept3,2024.docx [file pone.0311201.s010.docx]

**Response to Reviewers, Sept 3^rd^, 2024**

Thank you for the opportunity to address the reviewers’ recommended changes which are copied below in red.

Comment from the Editor: Please address the minor issues raised by Reviewers #4 and #5. 

Reviewer #4: Thanks to the Editorial Staff for the opportunity to review this revised article. It is an incredible piece of work. The scale of which was huge. I have read the article and I agree with the discussion.

Thank you.

I see on Page 4 (introduction) line 2 has an error in ' per 100,00 '). Two words are underlined on page 22 (results).

These changes have been made.

As a PEM physician I found the article relatively easy to comprehend. This is testament to the adjustments made in lieu of the previous reviewers and the balanced discussion.

We are pleased to hear our manuscript is easier to comprehend after our prior revision.

Reviewer #5: The authors have adequately addressed the reviewers' comments and queries.

As above, we are pleased to hear our prior revision has adequately addressed reviewers’ comments and queries.

The only additional comment I have is that I wonder about incorporation bias when looking at the values that diagnose pyogenic bacterial CAP, especially with regards to the inflammatory markers. If the members of the expert panel (i.e., the gold standard) believe that high inflammatory markers strongly point to a diagnosis of pyogenic bacterial CAP, and incorporate that into their evaluation, it would not be surprising that these markers predict what the reference standard considers pyogenic bacterial CAP. Perhaps a comment to that effect might be warranted in the limitations?

We have added “incorporation bias” as a limitation (line 365) in the Discussion.
